# Supplementary figures and images for: Mutations in Caenorhabditis elegans neuroligin-like glit-1, the apoptosis pathway and the calcium chaperone crt-1 increase dopaminergic neurodegeneration after 6-OHDA treatment
Source: PLoS Genet. 2018 Jan 18;14(1):e1007106. doi: 10.1371/journal.pgen.1007106 (PMC5773152; doi:10.1371/journal.pgen.1007106)

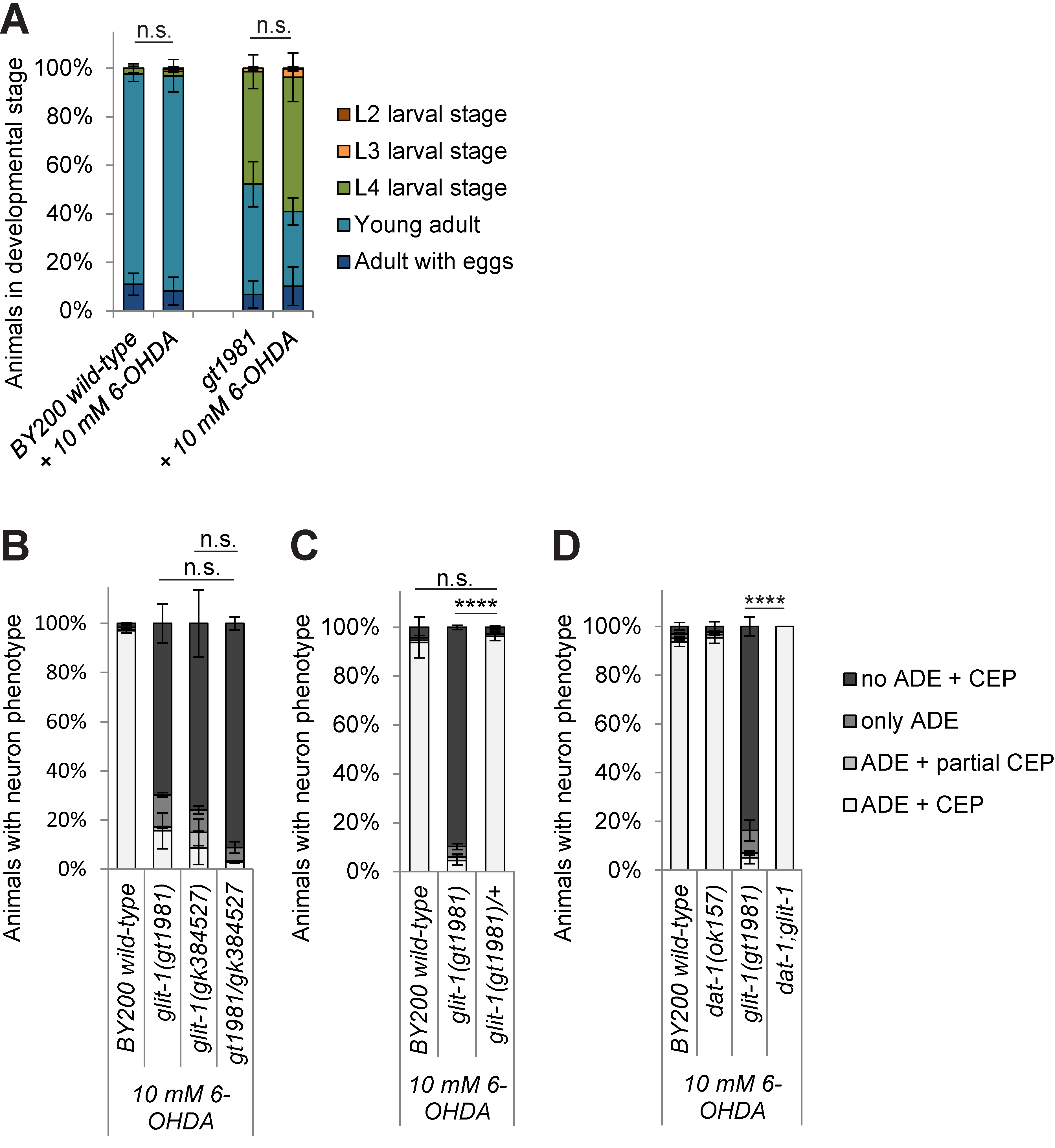

Supplement: S1 Fig — (A) Developmental stages of wild-type and gt1981 mutant L1 stage larvae 48 hours after treatment with and without 10 mM 6-OHDA. C. elegans develops via the L1, L2 (in red), L3 (in orange), L4 (green) and young adult stage (in light blue) into adults (in dark blue). Error bars = SEM of 3 biological replicates, each with 60–110 animals per treatment and strain. Total number of animals per condition n = 215–270 (n.s. p>0.05; G-Test). (B) Dopaminergic head neurons 72 hours after treatment with 10 mM 6-OHDA in BY200 wild-type animals, glit-1(gt1981) and glit-1(gk384527) homozygous mutants and glit-1/glit-1(gk384527) transheterozygote mutant animals. Error bars = SEM of 2–3 experiments, each with 60–200 animals per strain. Total number of animals per strain n = 270–340 (n.s. p>0.05; G-Test). (C) Dopaminergic head neurons 72 hours after treatment with 10 mM 6-OHDA in BY200 wild-type animals and glit-1(gt1981) homozygous and glit-1(gt1981)/+ heterozygous mutants. Error bars = SEM of 2 experiments, each with 30–105 animals per strain. Total number of animals per strain n = 65–210 (****p<0.0001, n.s. p>0.05; G-Test). (D) Effect of dat-1 mutation on dopaminergic neurodegeneration after treatment with 10 mM 6-OHDA. Error bars = SEM of 2 experiments, each with 30–110 animals per strain. Total number of animals per strain n = 100–400 (****p<0.0001; G-Test). (TIF) [file pgen.1007106.s001.tif]

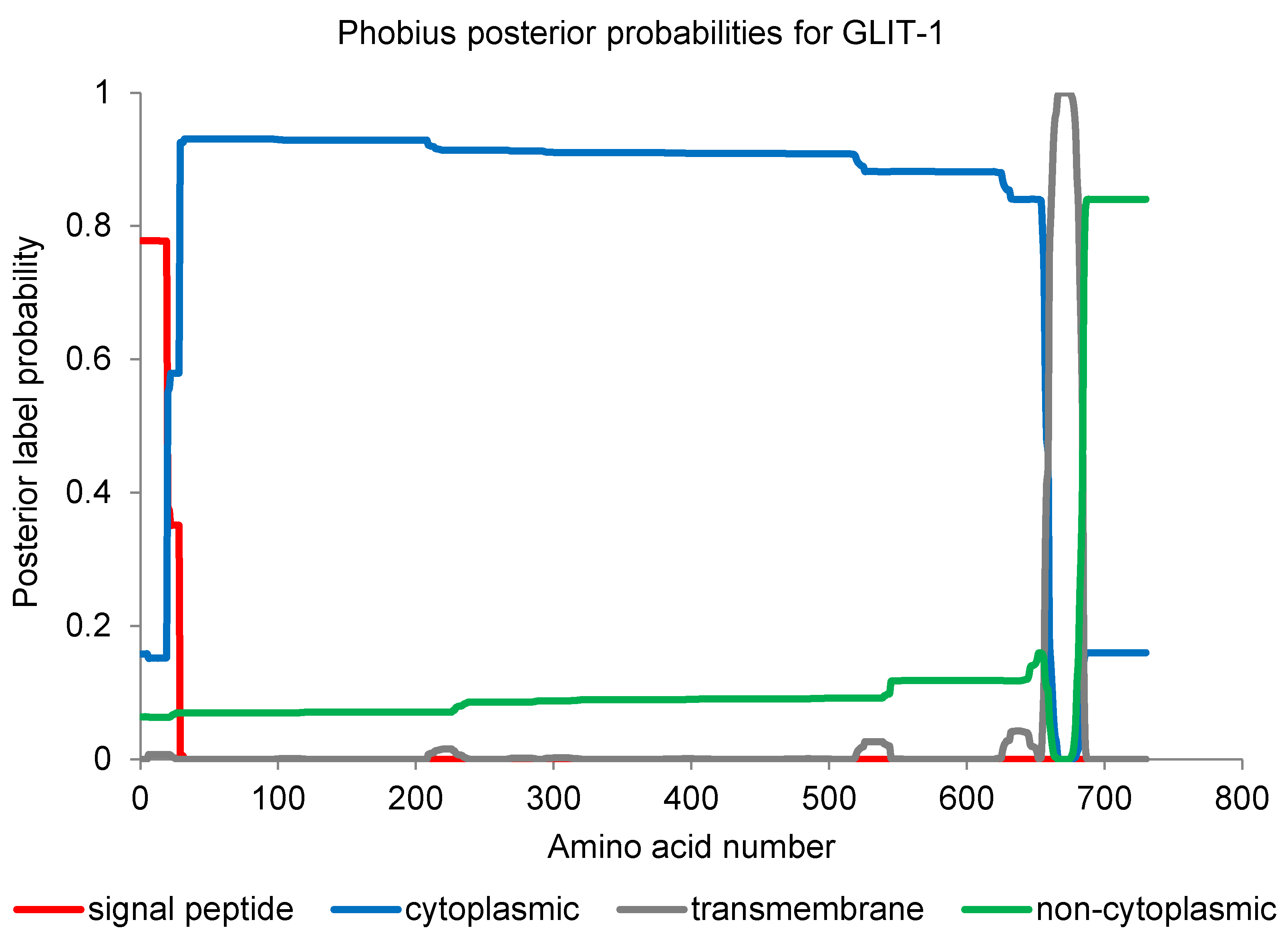

Supplement: S2 Fig — (A) Predicted probabilities for the occurrence of a signal peptide (in red), a transmembrane domain (in grey), a cytoplasmic part (in green) and a non-cytoplasmic part (in blue) as calculated with Phobius (http://phobius.sbc.su.se/). (TIF) [file pgen.1007106.s002.tif]

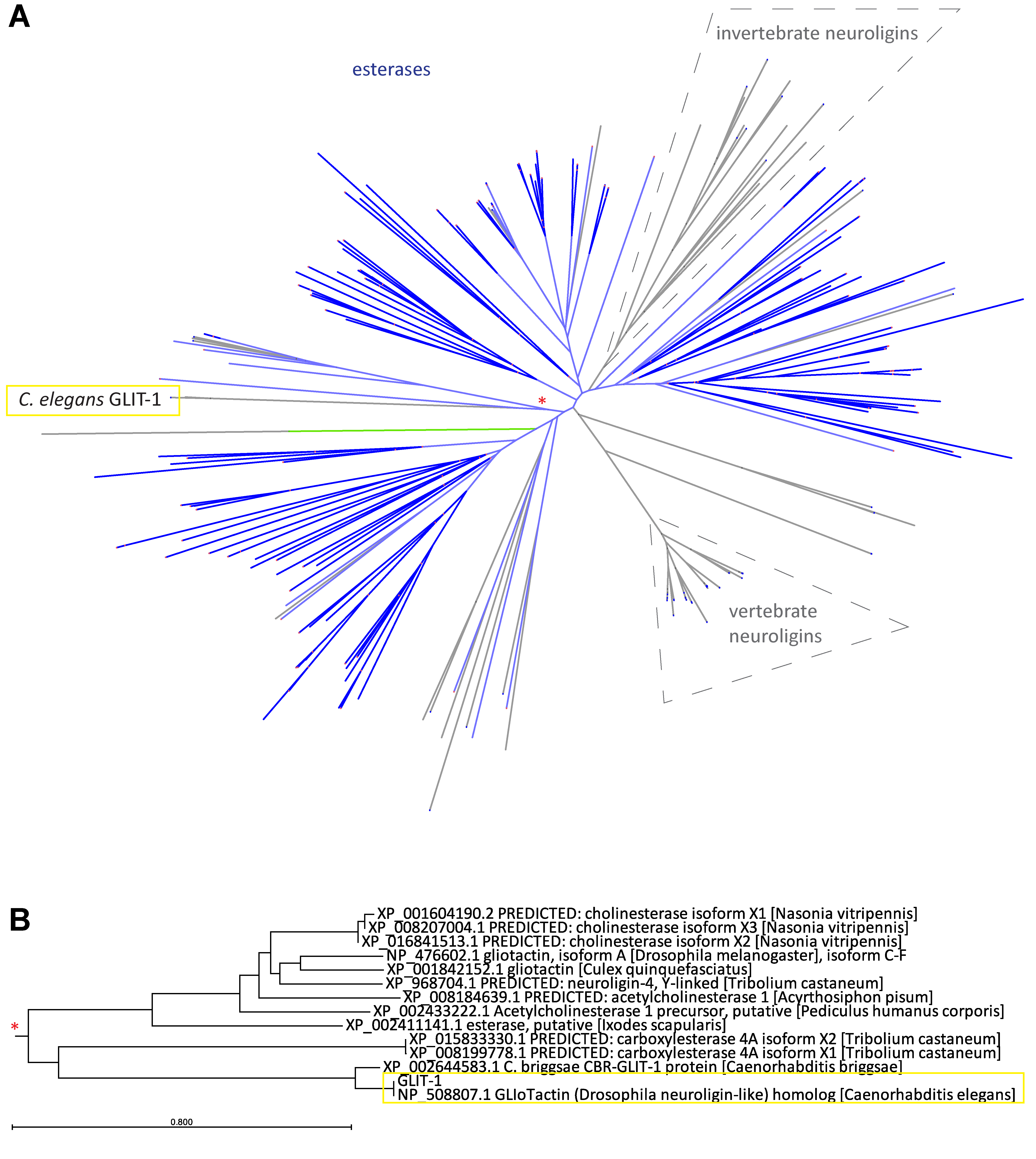

Supplement: S3 Fig — (A) Phylogenetic tree of GLIT-1 aligned to protein blast matches of selected species (listed below). The root of the tree is marked in green, esterases are marked in blue and vertebrate and invertebrate neuroligin groups are framed. The subtree containing GLIT-1 is indicated with a red asterisk. (B) Blow-up of the GLIT-1-containing subtree. Acyrthosiphon pisum (pea aphid), Caenorhabditis briggsae (nematode), Ciona intestinalis (sea squirt), Culex quinquefasciatus (southern house mosquito), Danio rerio (zebrafish), Drosophila melanogaster (fruit fly), Homo sapiens (human), Ixodes scapularis (tick), Mus musculus (house mouse), Nasonia vitripennis (parasitoid wasp), Pediculus humanus corporis (body louse), Schistosoma mansoni (parasitic trematode), Strongylocentrotus purpuratus (purple sea urchin), Tribolium castaneum (red flour beetle), Trichinella spiralis (parasitic nematode). (TIF) [file pgen.1007106.s003.tif]

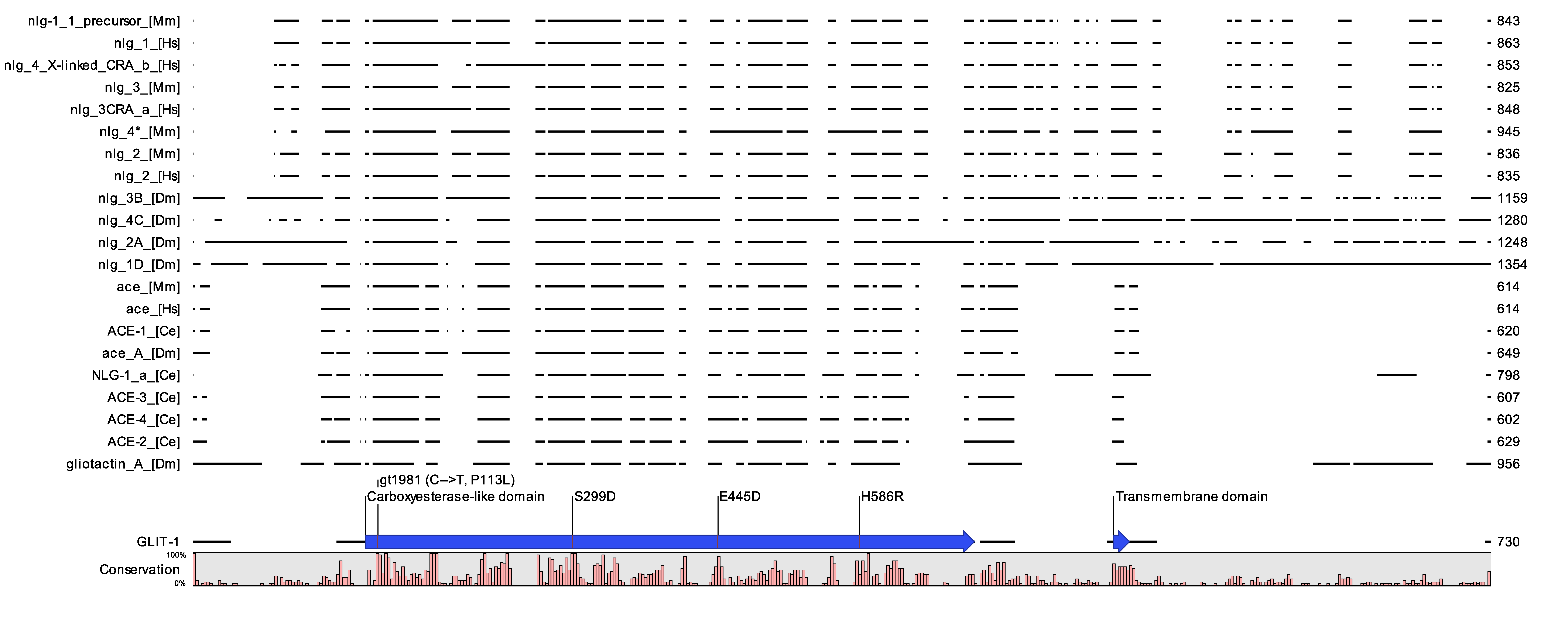

Supplement: S4 Fig — Alignment of acetylcholinesterases (ace) and neuroligins (nlg) from mouse (Mus musculus, Mm), human (Homo sapiens, Hs), fruit fly (Drosophila melanogaster, Dm), and C. elegans (Ce). Carboxyesterase-like domain and transmembrane domain are indicated with blue arrows on top of the GLIT-1 sequence. The conservation score is indicated with a bar graph at the bottom of the alignment. (TIF) [file pgen.1007106.s004.tif]

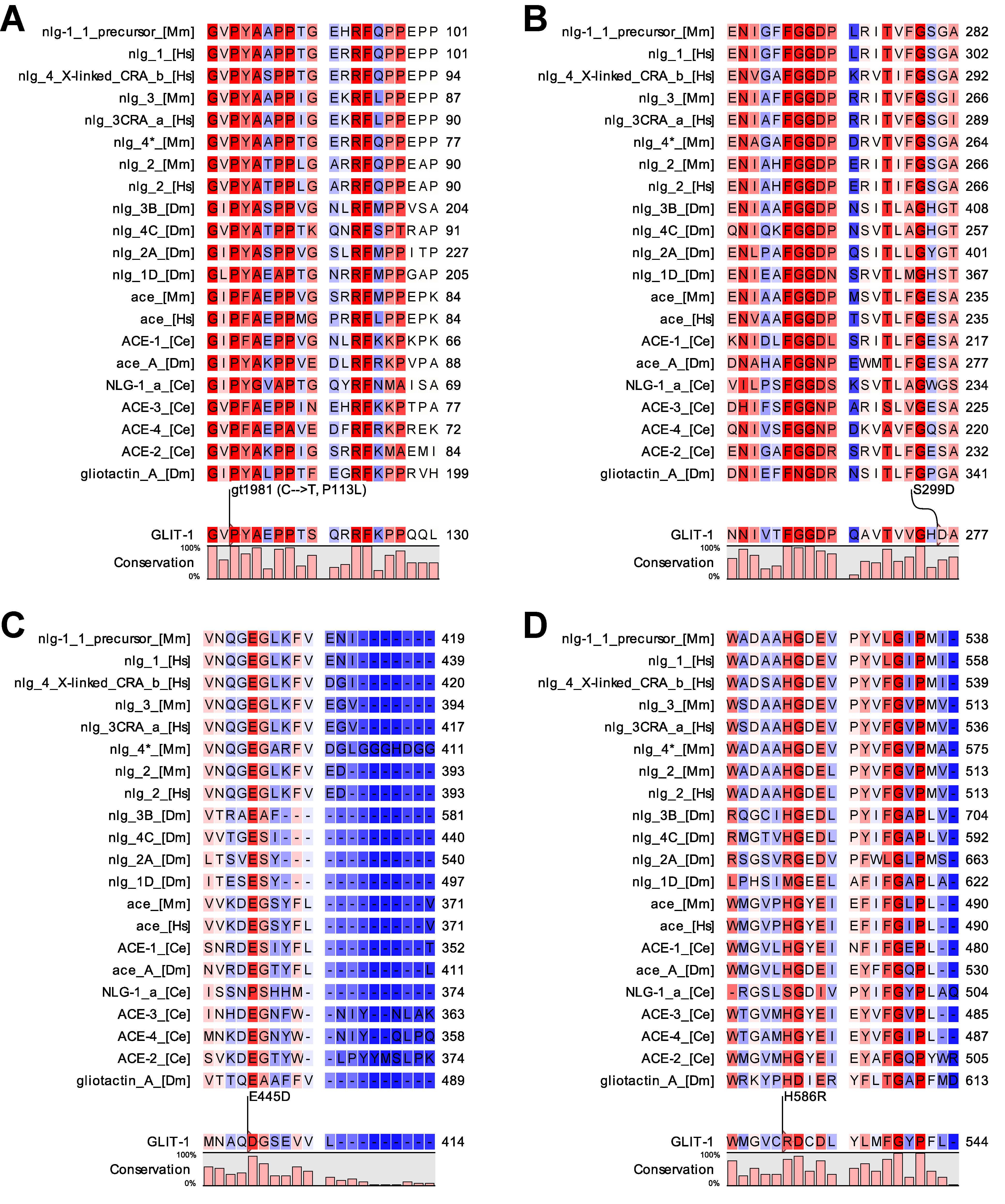

Supplement: S5 Fig — Magnification of alignment of acetylcholinesterases (ace) and neuroligins (nlg) from mouse (Mus musculus, Mm), human (Homo sapiens, Hs), fruit fly (Drosophila melanogaster, Dm), and C. elegans (Ce) to show the (A) proline residue mutated in glit-1(gt1981) (P113L) and the (B) serine, (C) histidine and (D) glutamate residues that form part of the catalytic triad of acetylcholinesterases. High conservation is highlighted in red and low conservation in blue. The conservation score is indicated with a bar graph at the bottom of the alignment. (TIF) [file pgen.1007106.s005.tif]

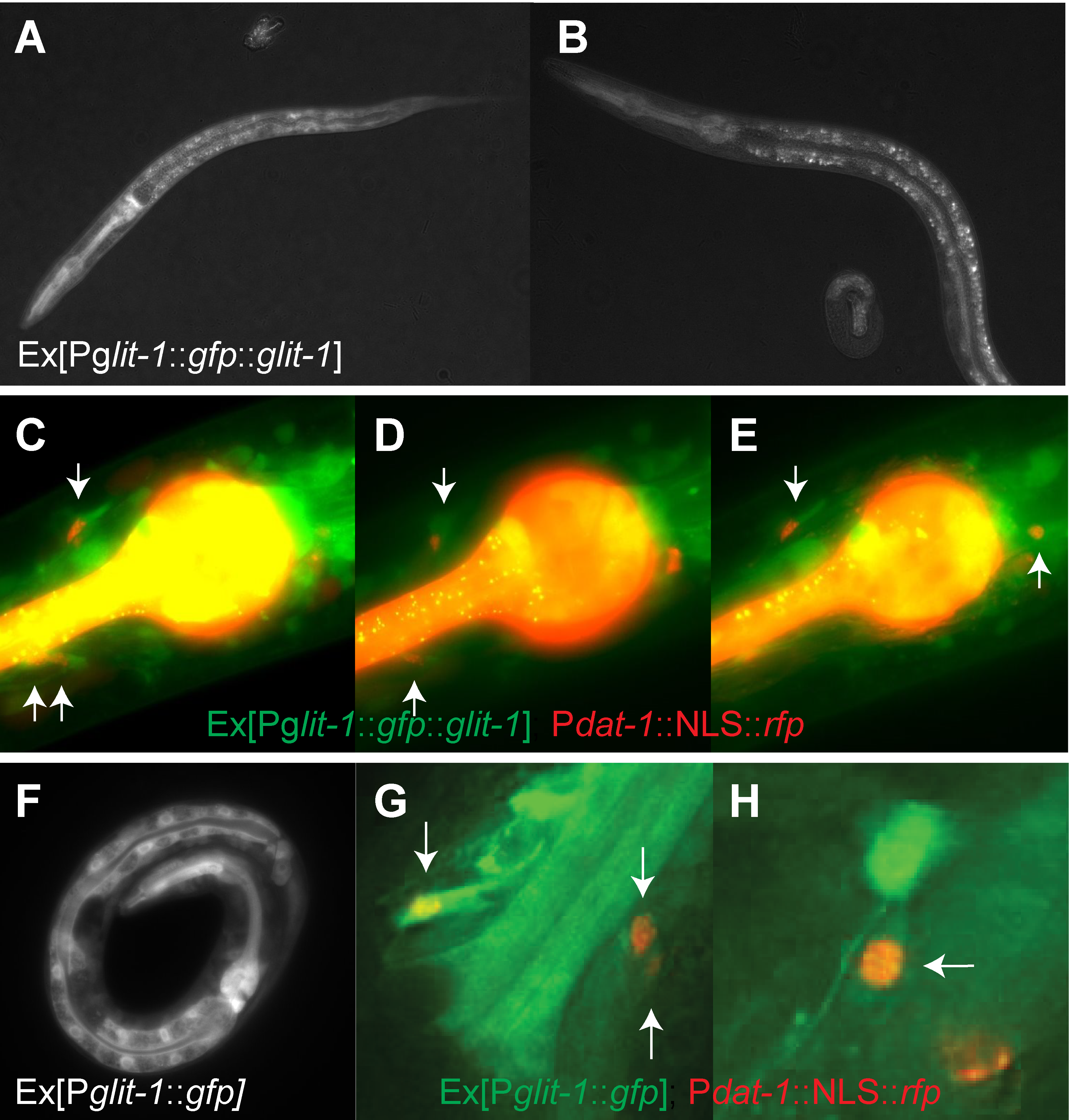

Supplement: S6 Fig — (A) L3 stage larva expressing Ex[Pglit-1::gfp::glit-1]. (B) Embryo and L4 stage larva expressing Ex[Pglit-1::gfp::glit-1]. (C)—(E) Head region of the L4 stage larva from Fig 2B–2F. The green channel shows expression of Ex[Pglit-1::gfp::glit-1]. The red channel shows expression of Is[Pdat-1::NLS::rfp;Pttx-3::mCherry] for labelling of dopaminergic neuron nuclei, as well as the pharynx muscle marker Ex[Pmyo-2::mCherry] and the body muscle marker Ex[Pmyo-3::mCherry] that were used for injections. (F) L1 stage larva expressing Ex[Pglit-1::gfp]. (G) and (H) Head region of adult animals. The green channel shows expression of Ex[Pglit-1::gfp]. The red channel shows expression of Is[Pdat-1::NLS::rfp;Pttx-3::mCherry] for labelling of dopaminergic neuron nuclei, as well as the pharynx muscle marker Ex[Pmyo-2::mCherry] and the body muscle marker Ex[Pmyo-3::mCherry] that were used for injections. (TIF) [file pgen.1007106.s006.tif]

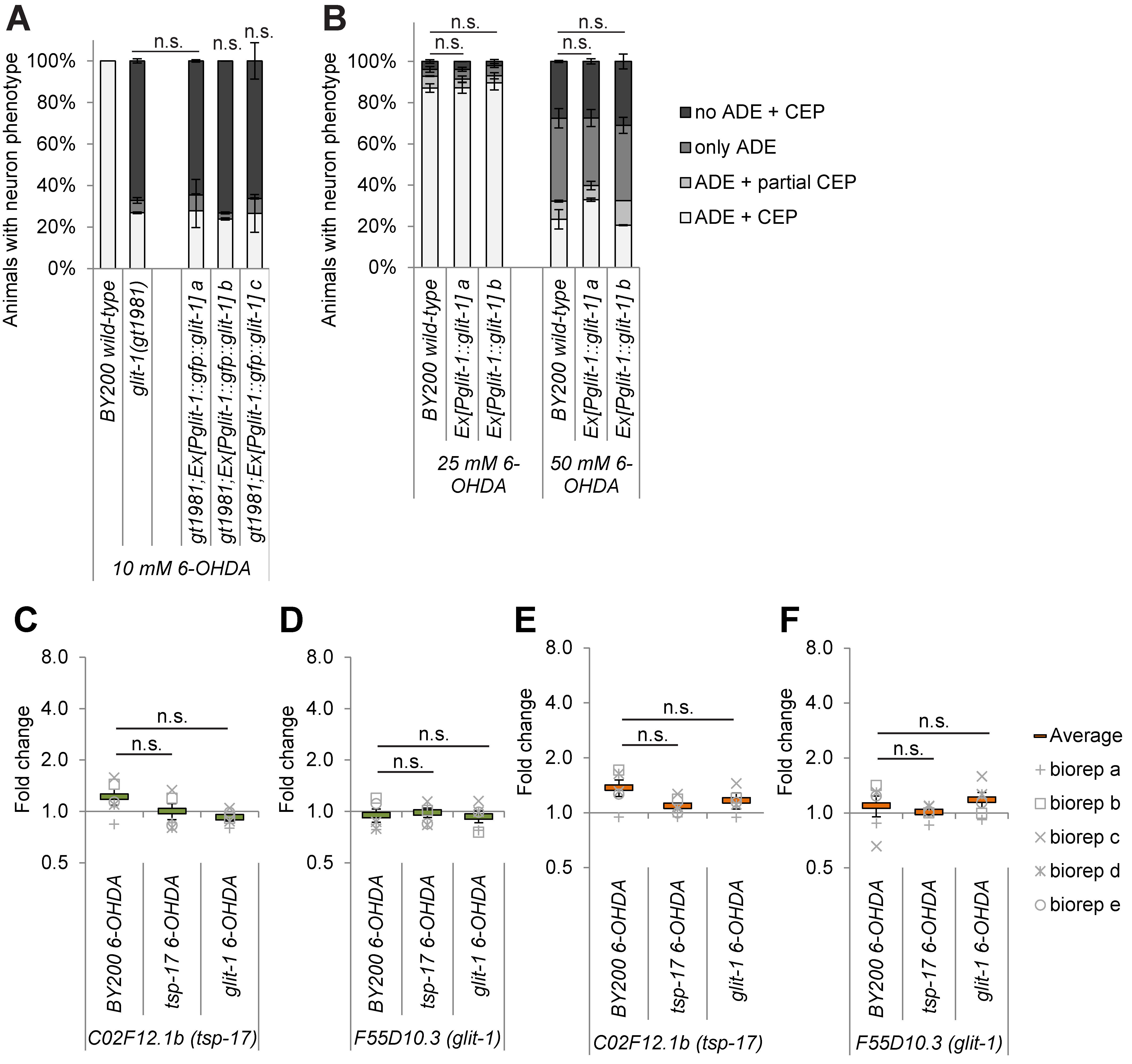

Supplement: S7 Fig — (A) Effects of Ex[Pglit-1::gfp::glit-1] expression on dopaminergic neurodegeneration in the glit-1 mutant after treatment with 10 mM 6-OHDA. Error bars = SEM of 2 biological replicates, each with 20–100 animals per strain and concentration. Total number of animals per strain n = 70–200 (n.s. p>0.05; G-Test). (B) Effect of multi-copy Ex (Pglit-1::glit-1] construct on dopaminergic neurodegeneration after treatment with 25 and 50 mM 6-OHDA. Error bars = SEM of 2 biological replicates, each with 80–110 animals per strain. Total number of animals per condition n = 185–215 (n.s. p>0.05; G-Test). (C) tsp-17 and (D) glit-1 mRNA levels in wild-type and mutant L1 stage larvae after 1 hour treatment with 10 mM 6-OHDA. The data are normalised to the control gene Y45F10D.4. The average and the respective values for 5 biological replicates (biorep a-e) are indicated. Error bars = SEM of 5 biological replicates. (E) tsp-17 and (F) glit-1 mRNA levels in wild-type and mutant L1 stage larvae after 1 hour treatment with 10 mM 6-OHDA. The data are normalised to the control gene pmp-3. The average and the respective values for 5 biological replicates (biorep a-e) are indicated. Error bars = SEM of 5 biological replicates. (TIF) [file pgen.1007106.s007.tif]

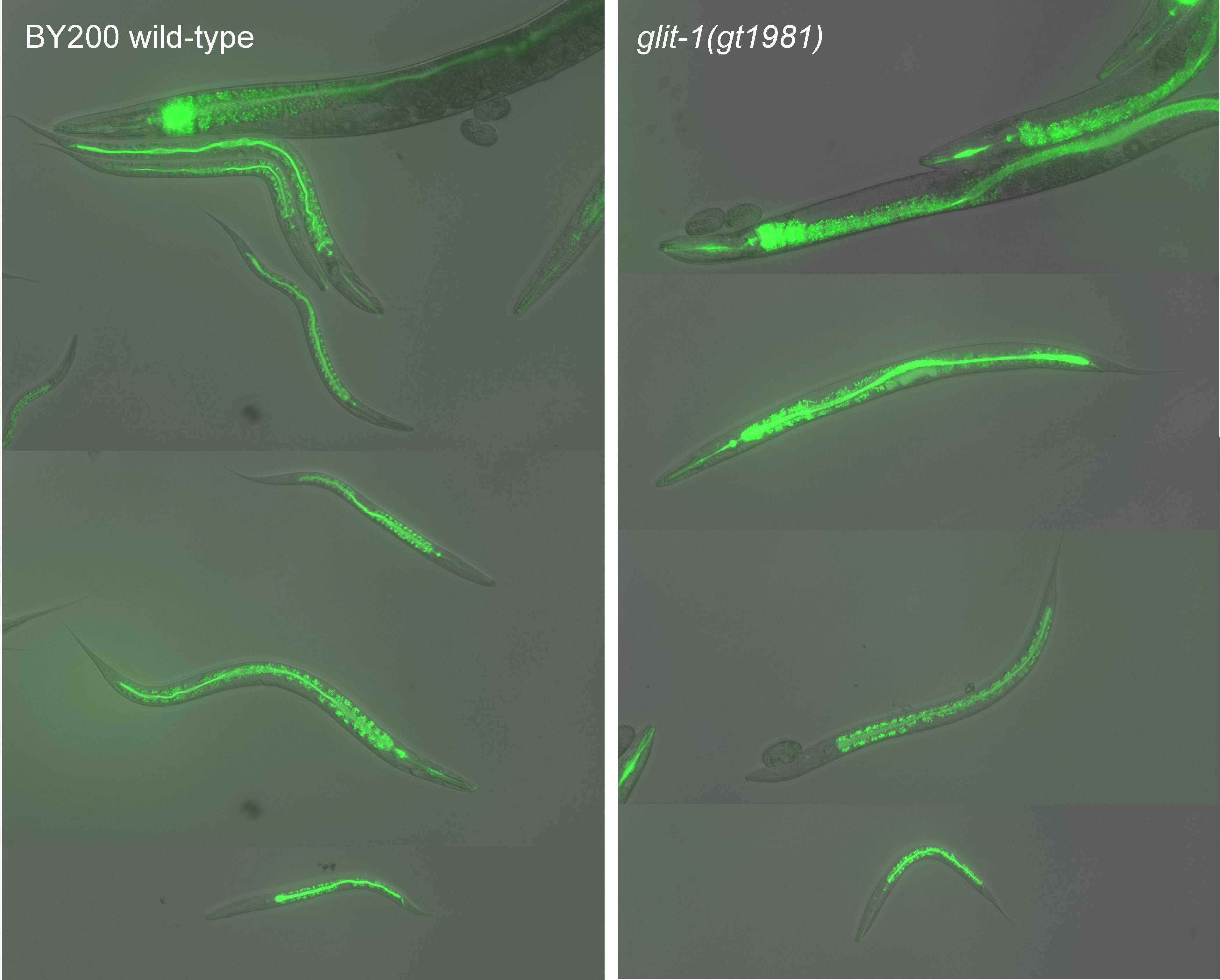

Supplement: S8 Fig — Overlay of fluorescence image (in green) and bright-field image (in grey) of wild-type and glit-1(gt1981) mutant animals after 3 hours of incubation in fluorescein sodium salt. (TIF) [file pgen.1007106.s008.tif]

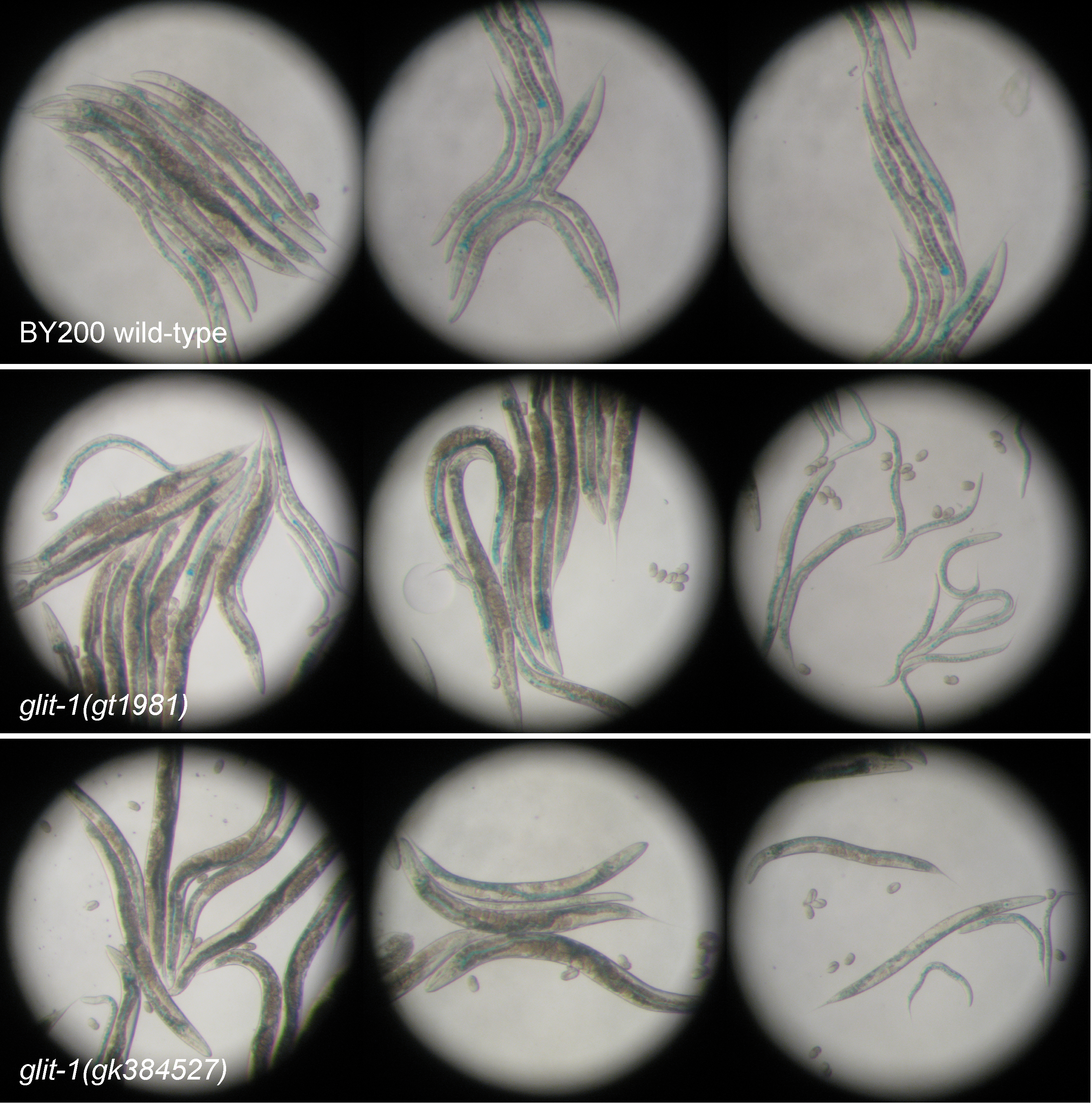

Supplement: S9 Fig — Wild-type and glit-1 mutant animals after 3 hours of incubation in Brilliant Blue FCF. (TIF) [file pgen.1007106.s009.tif]

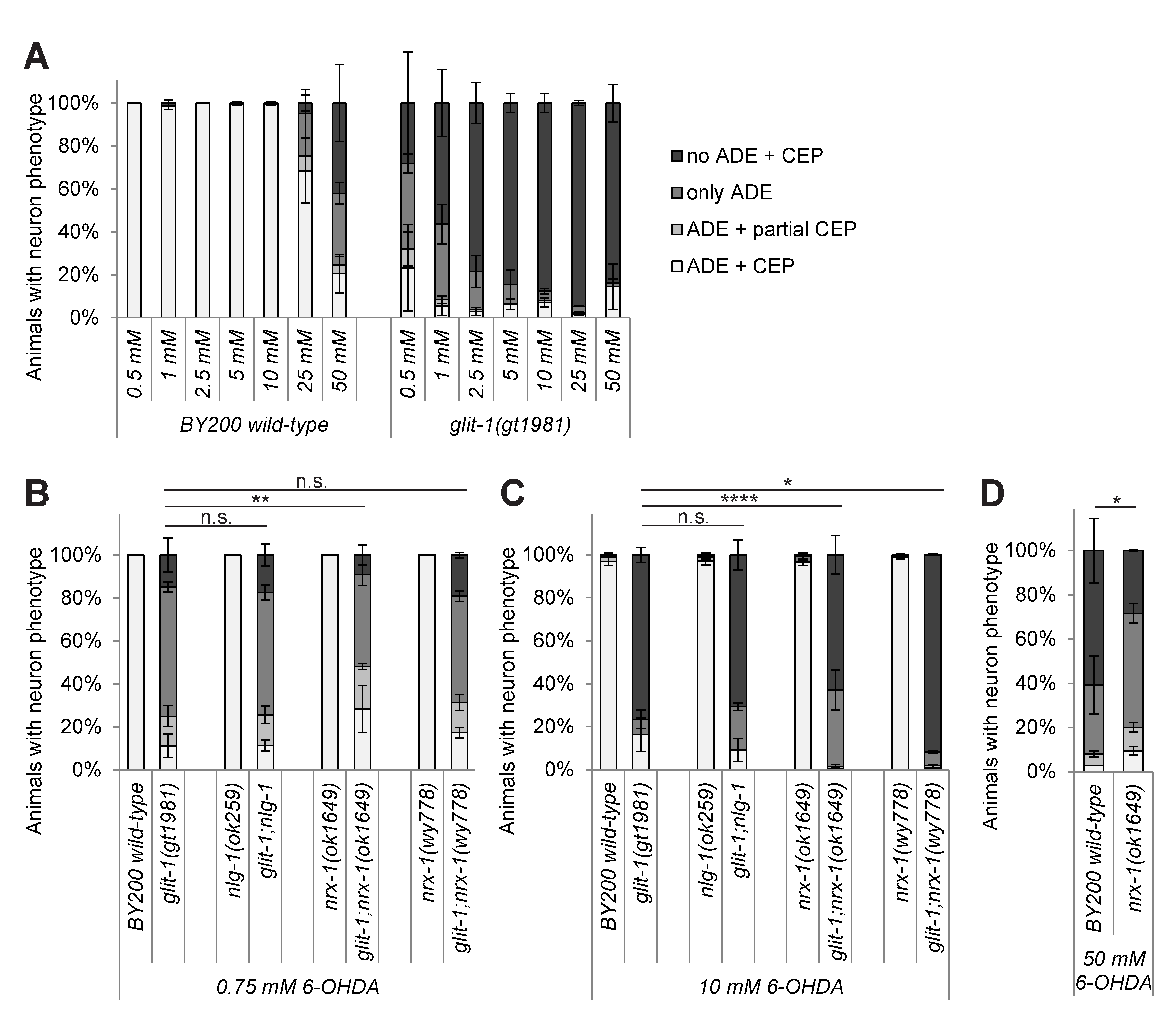

Supplement: S10 Fig — (A) Dopaminergic head neurons 72 hours after treatment with 10 mM 6-OHDA in wild-type and glit-1 mutant animals. Error bars = SEM of 1–2 experiments, each with 90–115 animals per strain. Total number of animals per strain n = 115–220. (B) Effects of nlg-1 and nrx-1 mutations on dopaminergic neurodegeneration after treatment with 0.75 mM, (C) 10 mM and (D) 50 mM 6-OHDA. Error bars = SEM of 2 biological replicates, each with 50–110 animals per strain and concentration. Total number of animals per condition n = 150–220 (****p<0.0001, **p<0.01, *p<0.05, n.s. p>0.05; G-Test). (TIF) [file pgen.1007106.s010.tif]

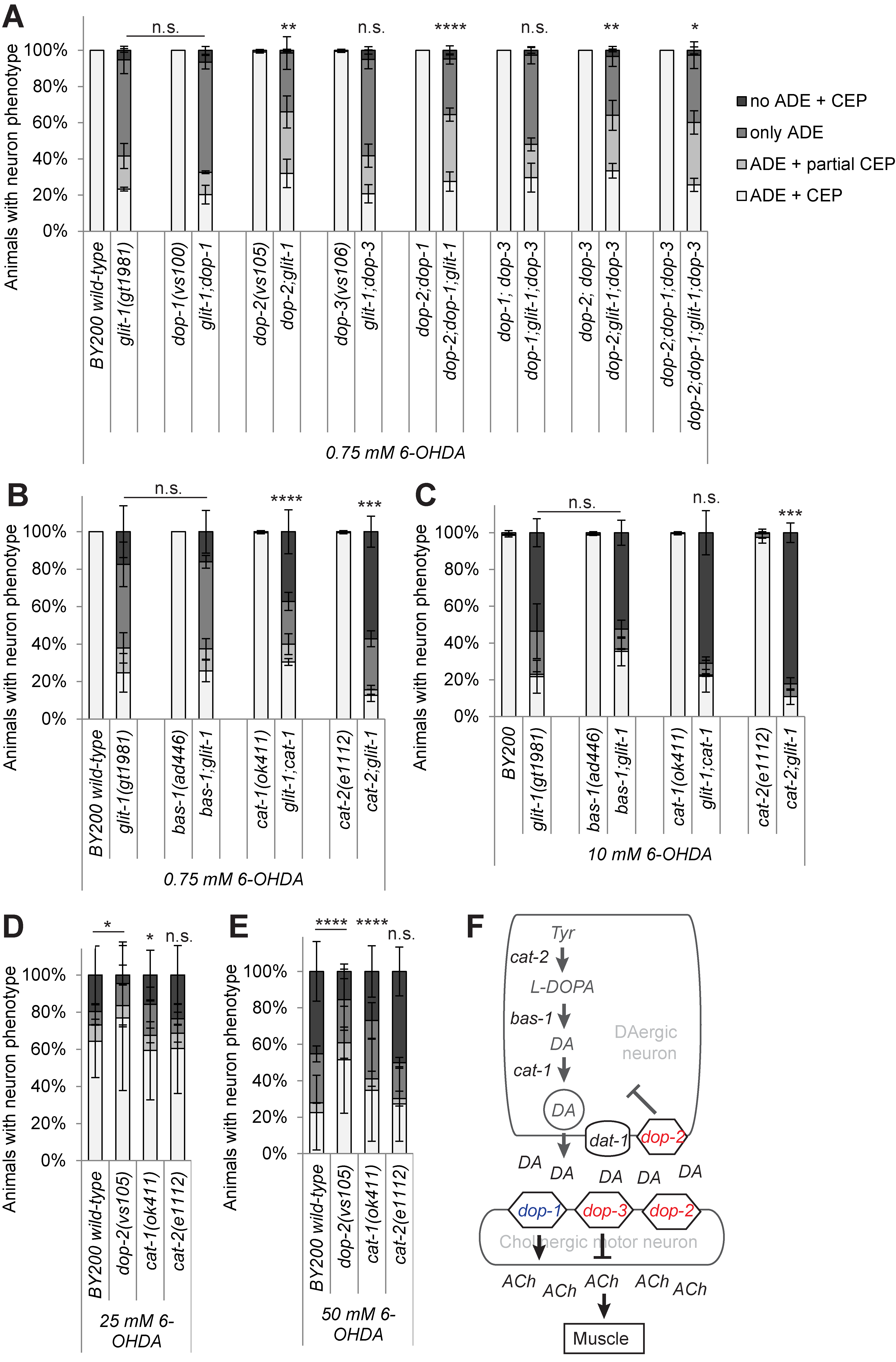

Supplement: S11 Fig — (A) Effect of dopamine receptor mutations on dopaminergic neurodegeneration after treatment with 0.75 mM 6-OHDA. Error bars = SEM of 3 biological replicates, each with 60–115 animals per strain and concentration. Total number of animals per condition n = 180–340 (****p<0.0001, **p<0.01, *p<0.05, n.s. p>0.05; G-Test comparing glit-1 mutant sensitivity to sensitivity of double and triple mutants). A significant p-value is only indicated if all or all but one replicate were found to be significant. (B) Effect of mutations in dopamine metabolism genes on dopaminergic neurodegeneration after treatment with 0.75 mM and (C) 10 mM 6-OHDA. Error bars = SEM of 3 biological replicates, each with 60–115 animals per strain and concentration. Total number of animals per condition n = 180–325 (****p<0.0001, ***p<0.001, n.s. p>0.05; G-Test comparing glit-1 to double mutants). (D) Effect of mutations in the dopamine receptor dop-2 and the dopamine metabolism genes cat-1 and cat-2 (abnormal catecholamine distribution) on dopaminergic neurodegeneration after treatment with 25 mM and (E) 50 mM 6-OHDA. Error bars = SEM of 3–4 biological replicates, each with 50–115 animals per strain and concentration. Total number of animals per condition n = 240–430 (****p<0.0001, *p<0.05, n.s. p>0.05; G-Test comparing BY200 wild-type to mutant animal data). A significant p-value indicated if all or all but one replicate were found to be significant. (F) Cartoon illustrating dopamine signalling cartoon (adapted from [23]). Dopamine synthesis in dopaminergic (DAergic) neurons starts with tyrosine (Tyr), which is converted to L-DOPA (L-3,4-dihydroxyphenylalanine) by the tyrosine hydroxylase CAT-2. L-DOPA is then converted to dopamine (DA) by the aromatic amino acid decarboxylase BAS-1 (biotenic amine synthesis related). Dopamine is packed into vesicles by the vesicular monoamine transporter CAT-1. Postsynaptic dopamine signalling is stimulated by the D1-like dopamine receptor DOP-1 and inhibited by the [file pgen.1007106.s011.tif]

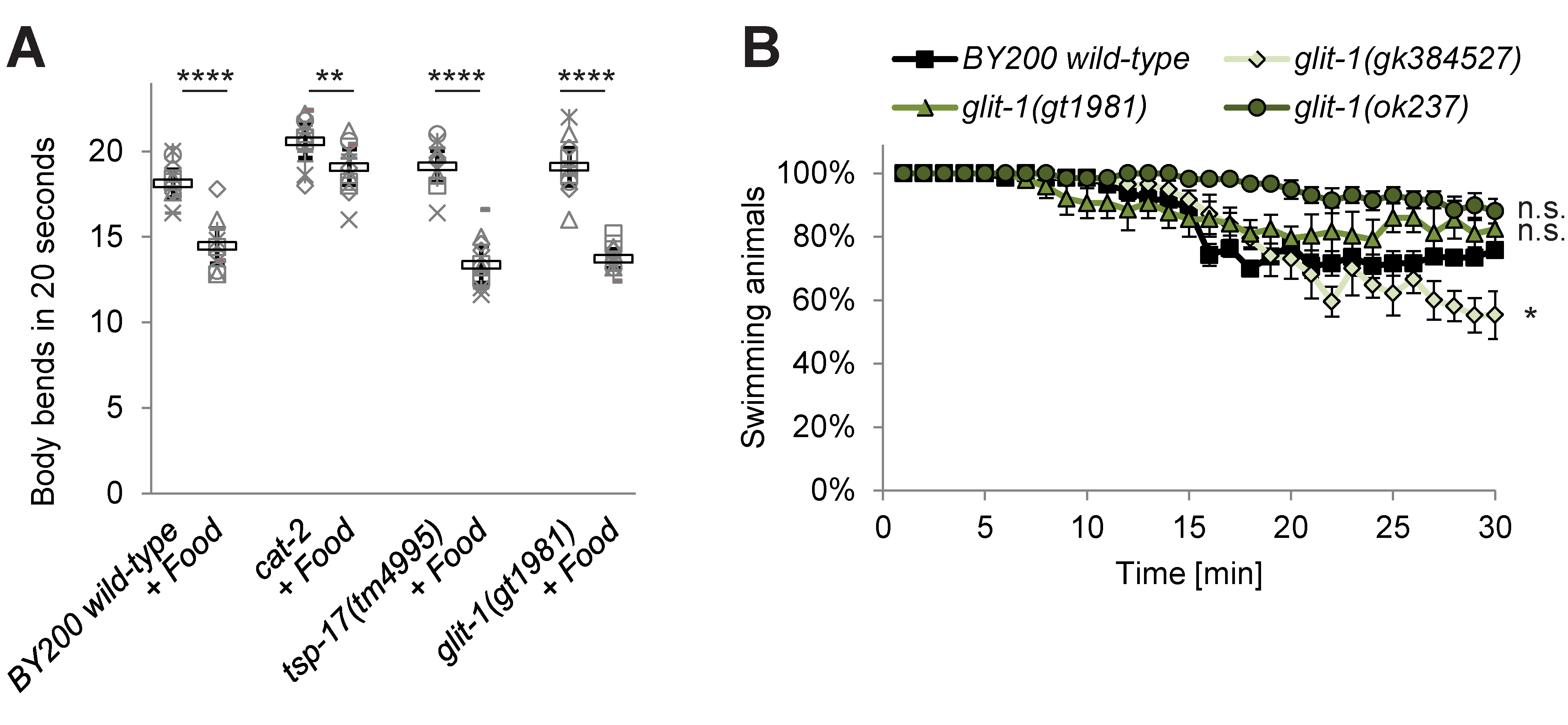

Supplement: S12 Fig — (A) Animal speed (measured in body bends/20s) on plates without and with food (‘+Food’). Indicated is the average speed per young adult animal (grey symbols) and the average speed per strain (black bar). cat-2 (abnormal catecholamine distribution) is a tyrosine hydroxylase mutant and defective in dopamine synthesis. Error bars = SEM of 2 biological replicates, each with 6 animals per strain and state. Total number of animals per condition n = 12 (****p<0.0001, **p<0.0001; two-tailed t-test). (B) Percentage of swimming L4 stage animals. Error bars = SEM of 3–4 biological replicates with 12–18 animals per strain. Total number of animals per strain n = 50–60 (*p<0.05, n.s. p>0.05; two-tailed t-test comparing wild-type and mutant data at 30 minute time point). (TIF) [file pgen.1007106.s012.tif]

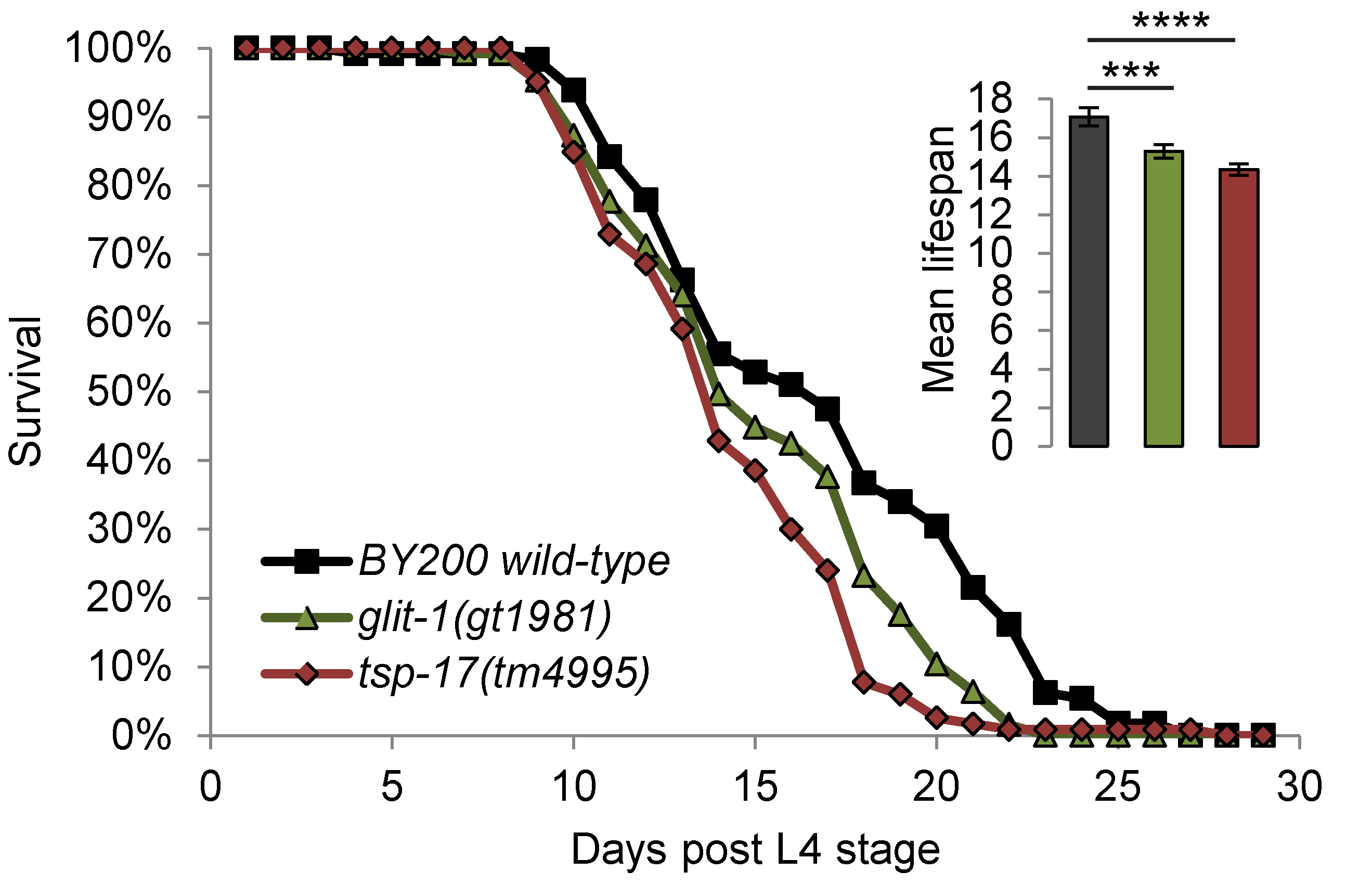

Supplement: S13 Fig — Lifespan data for second biological replicate including 88–109 animals per strain. The inset shows the mean lifespan with the error bars depicting the standard error (****p<0.0001, ***p<0.001; Bonferroni-corrected; Log-Rank Test). (TIF) [file pgen.1007106.s013.tif]

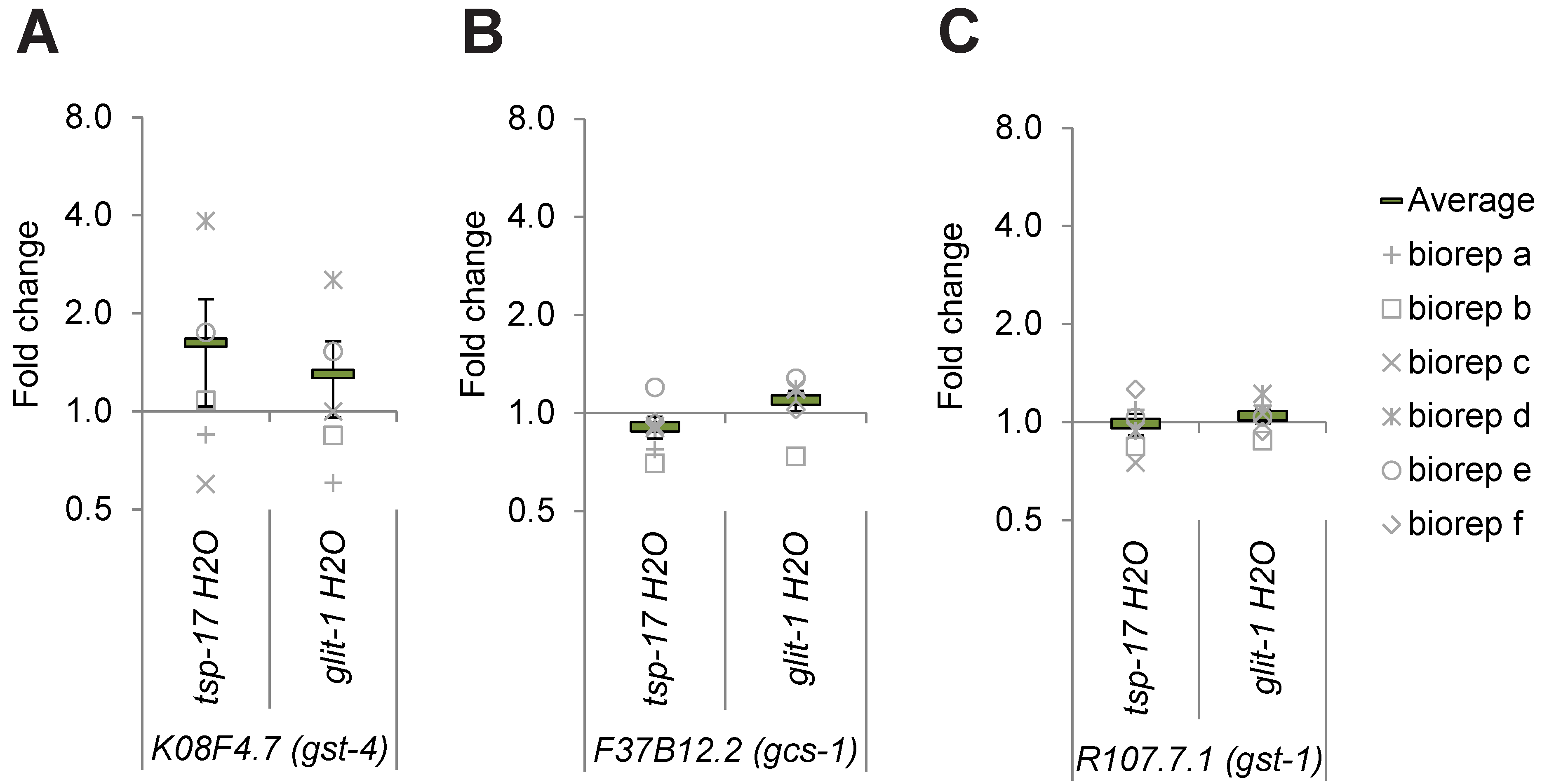

Supplement: S14 Fig — (A) gst-4, (B) gcs-1 and (C) gst-1 mRNA levels in mutant L1 stage larvae under control conditions (treatment with H2O instead of 6-OHDA). (A)-(C) The fold change is calculated based on mRNA levels in wild-type L1 stage larvae under control conditions. The data are normalised to the control gene Y45F10D.4 and the average and the respective values for 5–6 biological replicates (biorep a-f) are indicated. Error bars = SEM of 5–6 biological replicates. (TIF) [file pgen.1007106.s014.tif]

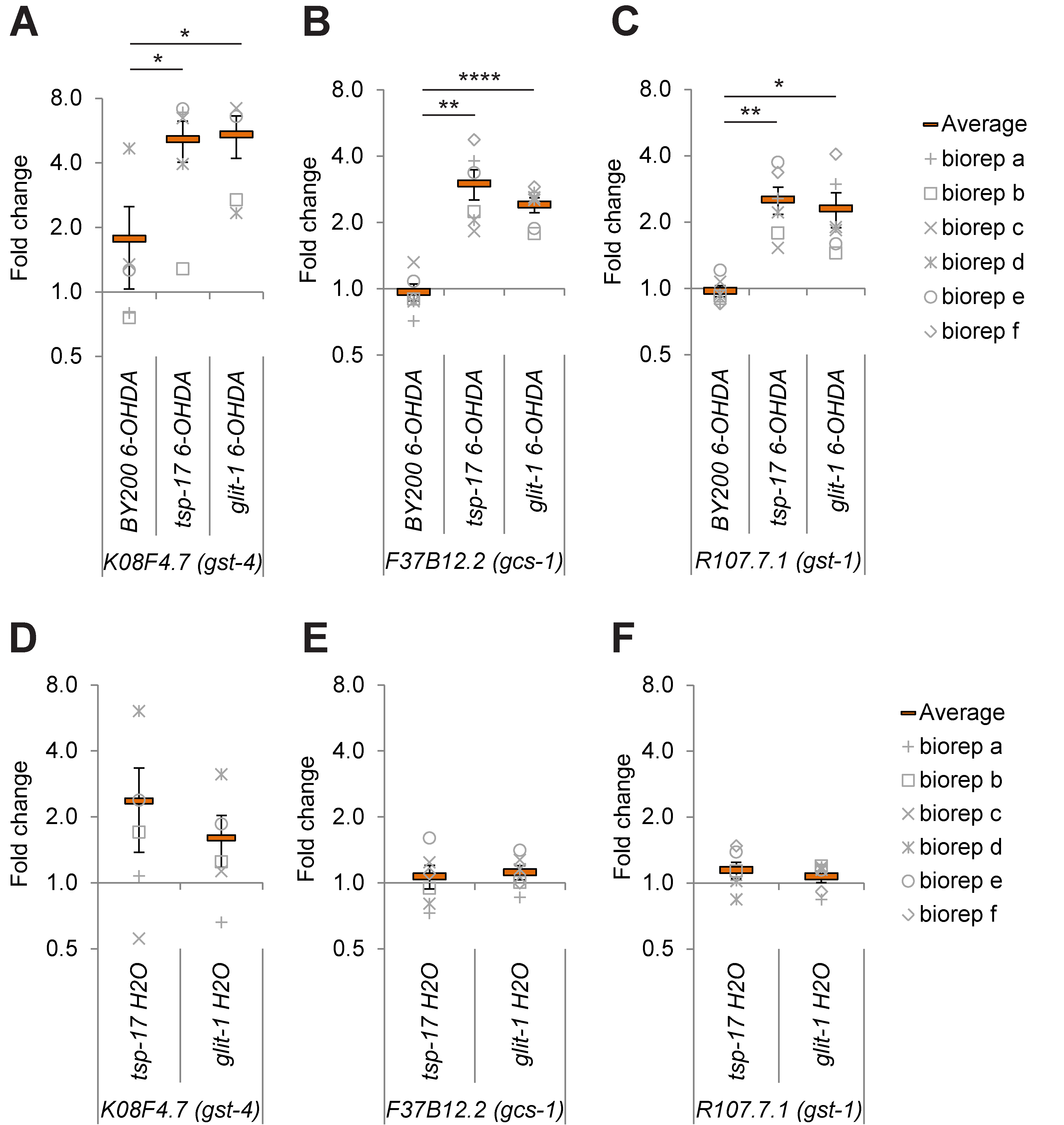

Supplement: S15 Fig — (A) gst-4, (B) gcs-1 and (C) gst-1 mRNA levels in wild-type and mutant L1 stage larvae after 1 hour treatment with 10 mM 6-OHDA. (D) gst-4, (E) gcs-1 and (F) gst-1 mRNA levels in mutants L1 stage larvae under control conditions (treatment with H2O instead of 6-OHDA). The fold change is calculated based on mRNA levels in wild-type L1 stage larvae under control conditions. (A)-(F) The data are normalised to the control gene pmp-3 and the average and the respective values for 5–6 biological replicates (biorep a-f) are indicated. Error bars = SEM of 5–6 biological replicates (****p<0.0001, **p<0.01, *p<0.05; two-tailed t-test). (TIF) [file pgen.1007106.s015.tif]

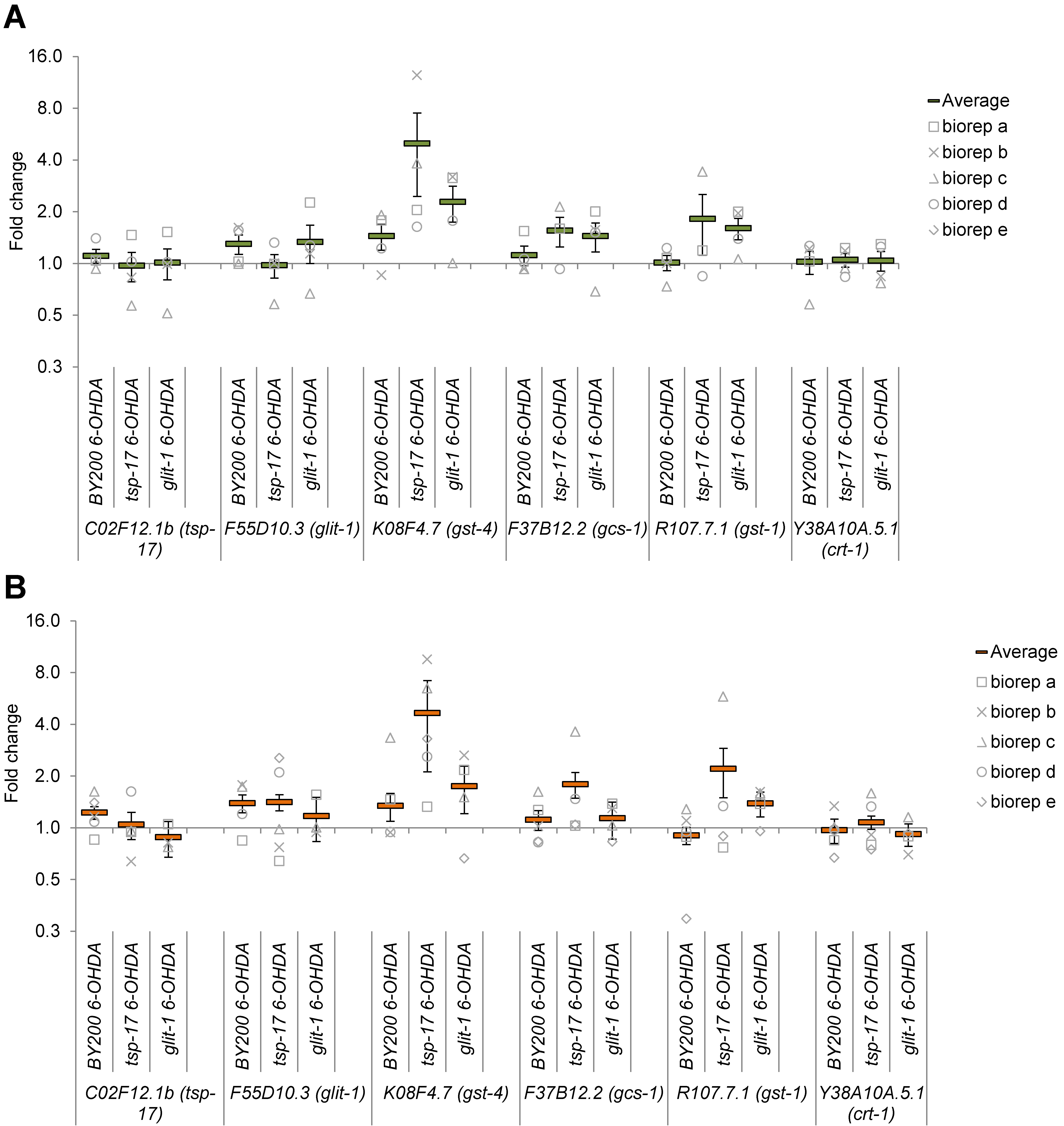

Supplement: S16 Fig — (A) tsp-17, glit-1, gst-4, gcs-1, gst-1 and crt-1 mRNA levels in wild-type and mutant L1 stage larvae after 1 hour treatment with 3 mM 6-paraquat. The data are normalised to the control gene Y45F10D.4. (B) tsp-17, glit-1, gst-4, gcs-1, gst-1 and crt-1 mRNA levels in wild-type and mutant L1 stage larvae after 1 hour treatment with 3 mM 6-paraquat. The data are normalised to the control gene pmp-3. (A) and (B) The average and the respective values for 3–5 biological replicates (biorep a-e) are indicated. Error bars = SEM of 3–5 biological replicates. (TIF) [file pgen.1007106.s016.tif]

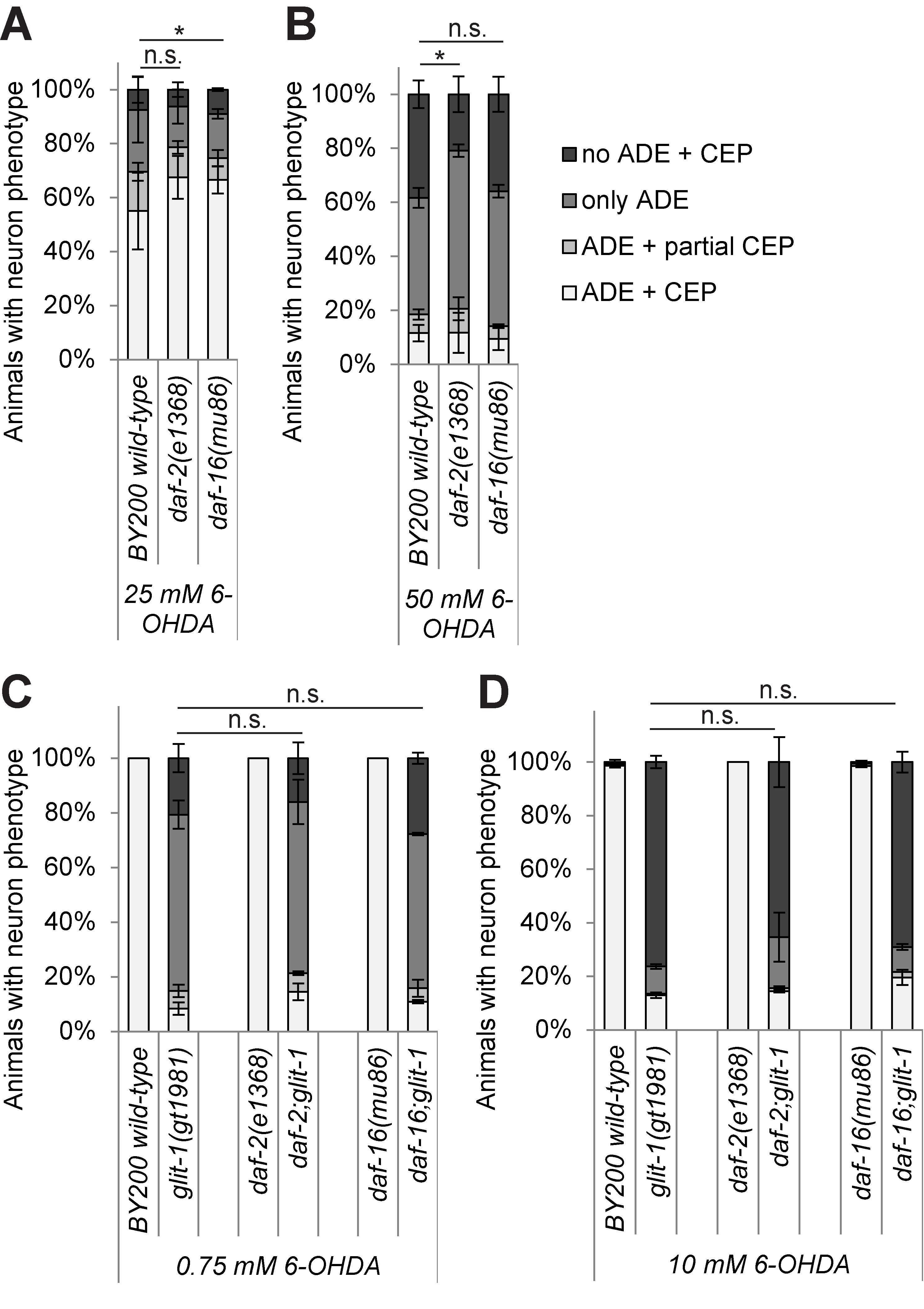

Supplement: S17 Fig — (A) Effect of daf-2 and daf-16 mutation on dopaminergic neurodegeneration after treatment with 25 mM and (B) 50 mM 6-OHDA. Error bars = SEM of 3 biological replicates with 85–135 scored animals per strain. Total number of animals per strain n = 320–360 (*p<0.05, n.s. p>0.05; G-Test). (C) Effect of daf-2 and daf-16 mutation on dopaminergic neurodegeneration in wild-type and glit-1 single and double mutant animals after treatment with 0.75 mM and (D) 10 mM 6-OHDA. Error bars = SEM of 2–3 biological replicates with 80–130 scored animals per condition. Total number of animals per strain n = 200–340 (n.s. p>0.05; G-Test). (TIF) [file pgen.1007106.s017.tif]

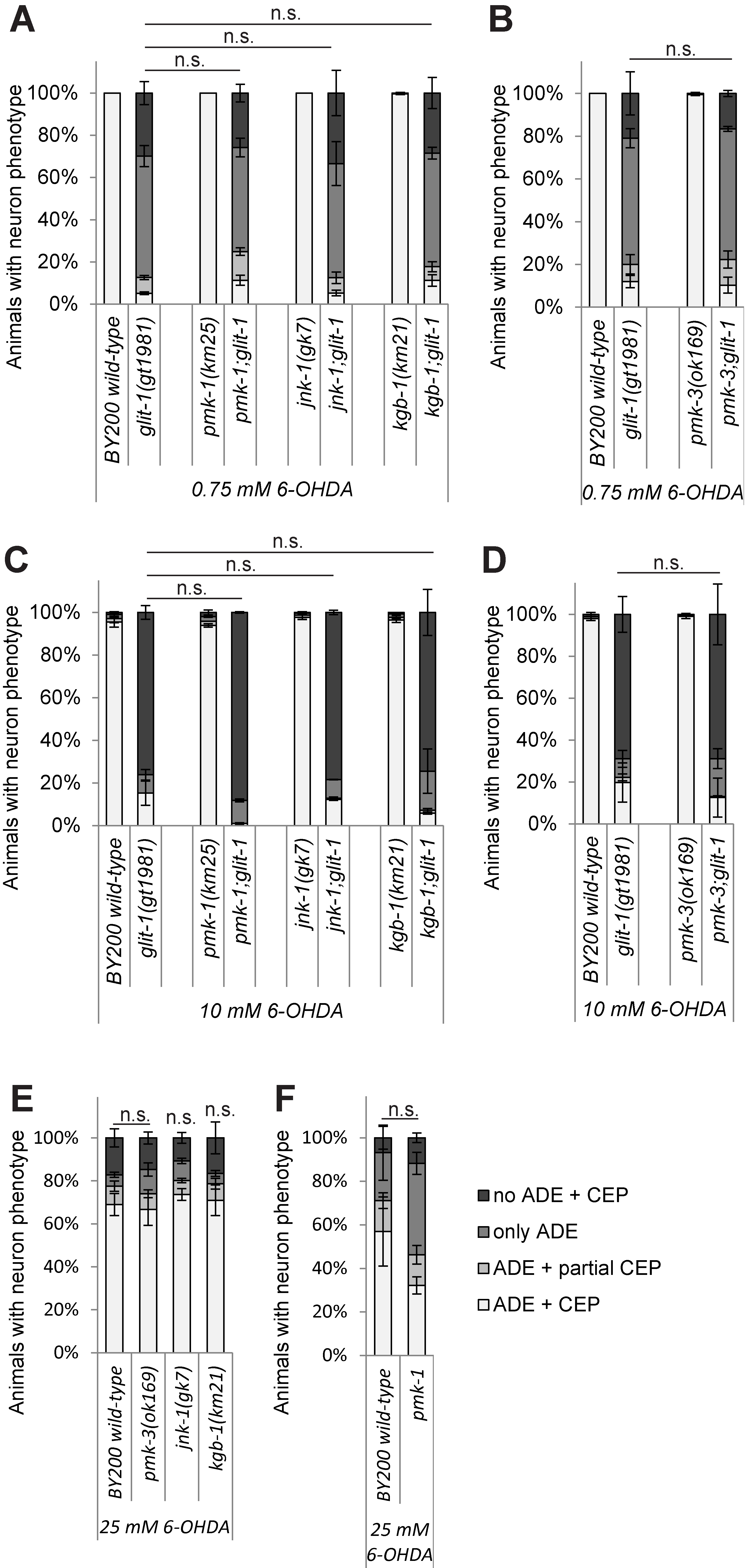

Supplement: S18 Fig — (A) Effect of p38 and JNK stress response pathway mutations on dopaminergic neurodegeneration after treatment with 0.75 mM 6-OHDA. Error bars = SEM of 3 biological replicates, each with 100–120 animals per strain. Total number of animals per strain n = 300–330 (n.s. p>0.05; G-Test). (B) Effect of pmk-3 mutation on dopaminergic neurodegeneration after treatment with 0.75 mM 6-OHDA. Error bars = SEM of 2–3 biological replicates, each with 50–110 animals per strain. Total number of animals per strain n = 160–300 (n.s. p>0.05; G-Test). (C) Effect of p38 and JNK stress response pathway mutations on dopaminergic neurodegeneration after treatment with 10 mM 6-OHDA. Error bars = SEM of 2–3 biological replicates, each with 40–105 animals per strain. Total number of animals per strain n = 195–315 (n.s. p>0.05; G-Test). (D) Effect of pmk-3 mutation on dopaminergic neurodegeneration after treatment with 10 mM 6-OHDA. Error bars = SEM of 2–3 biological replicates, each with 30–100 animals per strain. Total number of animals per strain n = 140–270 (n.s. p>0.05; G-Test). (E) Effect of p38 and JNK stress response pathway mutations on dopaminergic neurodegeneration after treatment with 25mM 6-OHDA. Error bars = SEM of 3 biological replicates, each with 100–110 animals per strain. Total number of animals per strain n = 310–330 (n.s. p>0.05; G-Test). (F) Effect of pmk-1 mutation on dopaminergic neurodegeneration after treatment with 25 mM 6-OHDA. Error bars = SEM of 3 biological replicates, each with 100–135 animals per strain. Total number of animals per strain n = 350–360 (n.s. p>0.05; G-Test). (TIF) [file pgen.1007106.s018.tif]

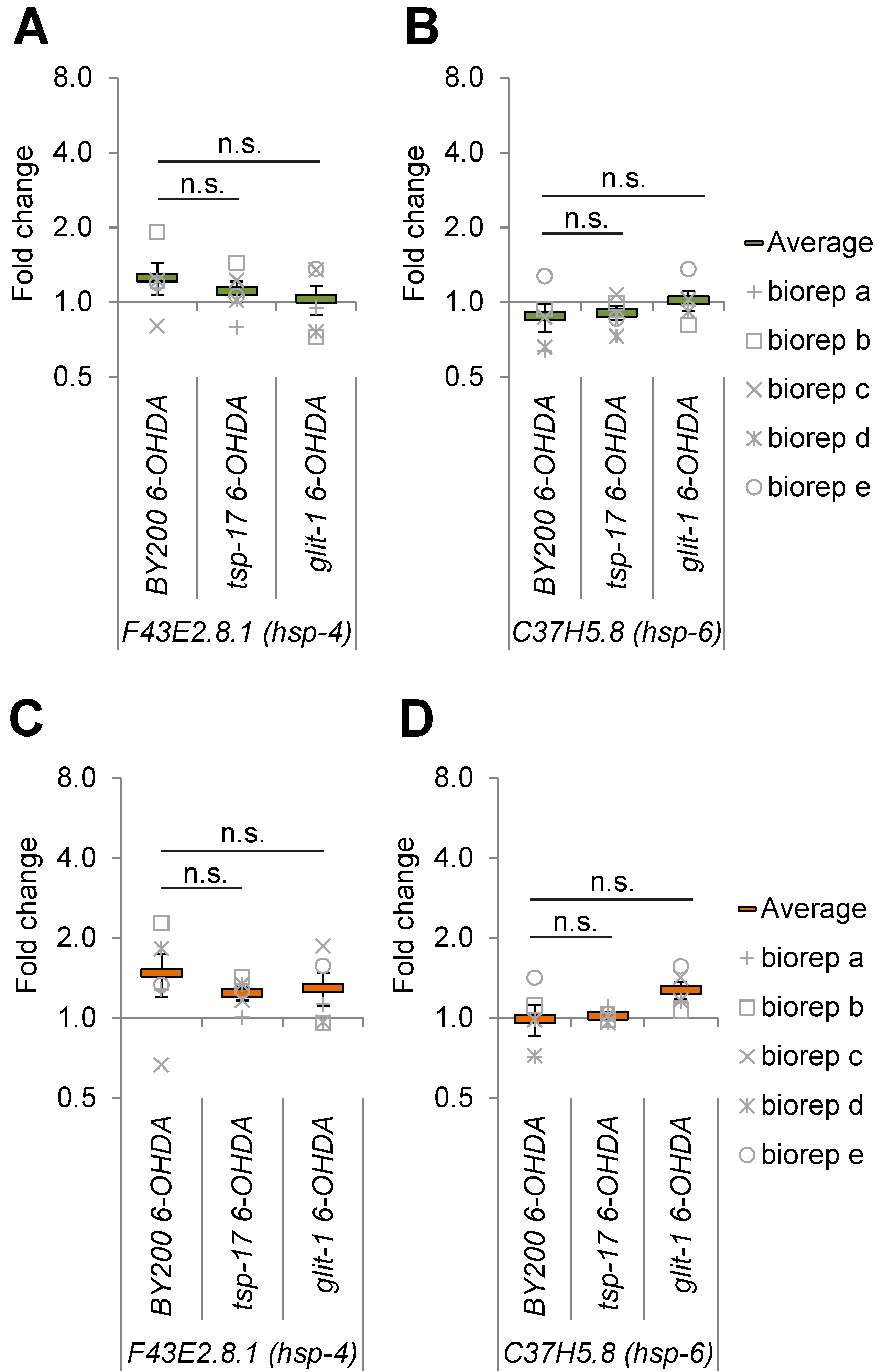

Supplement: S19 Fig — (A) hsp-4 and (B) hsp-6 mRNA levels in wild-type and mutant L1 stage larvae after 1 hour treatment with 10 mM 6-OHDA. The data are normalised to the control gene Y45F10D.4. The average and the respective values for 5 biological replicates (biorep a-e) are indicated. Error bars = SEM of 5 biological replicates. (C) hsp-4 and (D) hsp-6 mRNA levels in wild-type and mutant L1 stage larvae after 1 hour treatment with 10 mM 6-OHDA. The data are normalised to the control gene pmp-3. The average and the respective values for 5 biological replicates (biorep a-e) are indicated. Error bars = SEM of 5 biological replicates. (TIF) [file pgen.1007106.s019.tif]

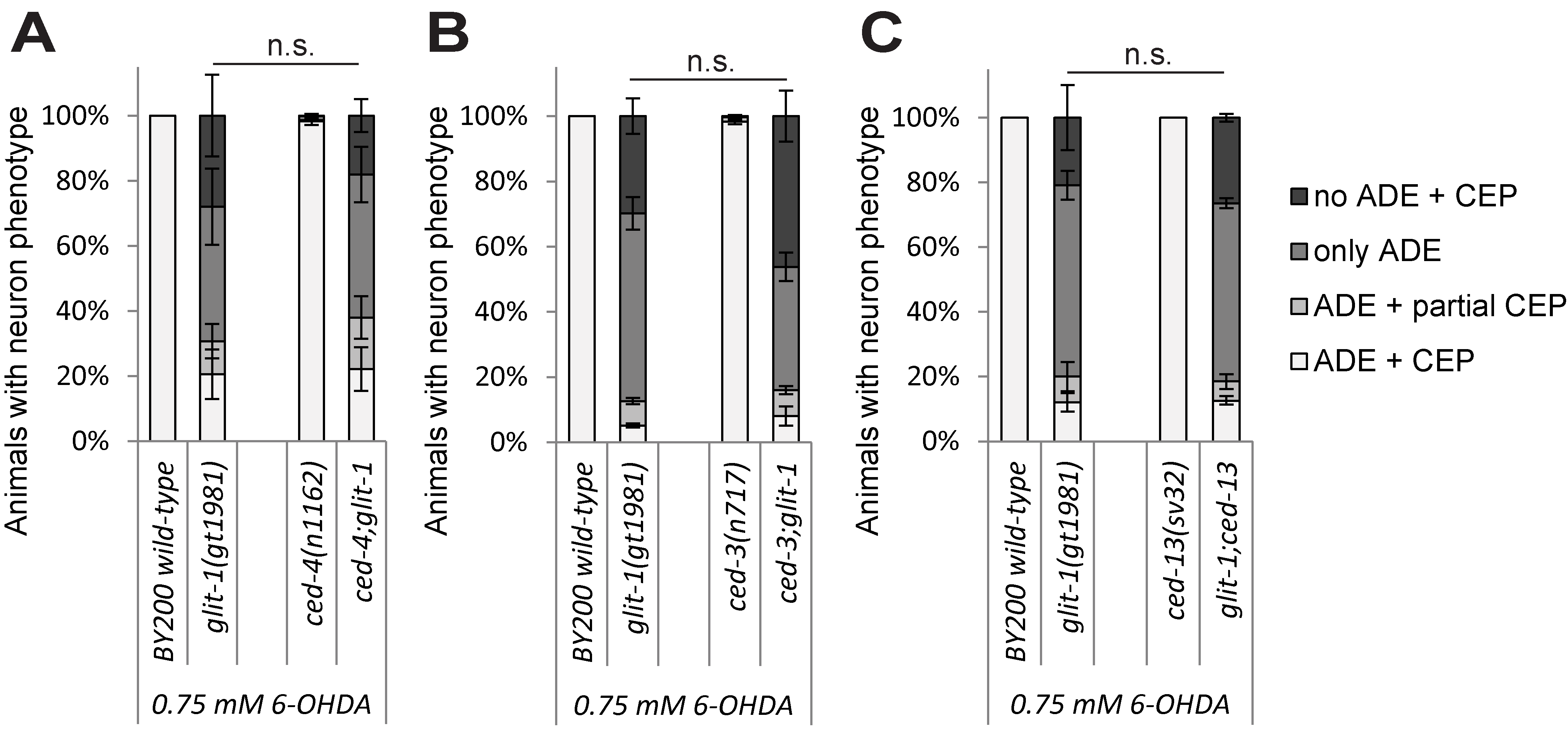

Supplement: S20 Fig — (A) Effect of ced-4 mutation on dopaminergic neurodegeneration after treatment with 0.75 mM 6-OHDA. Error bars = SEM of 5 biological replicates, each with 60–115 animals per strain and concentration. Total number of animals per condition n = 370–530 (n.s. p>0.05; G-Test). (B) Effect of ced-3 mutation on dopaminergic neurodegeneration after treatment with 0.75 mM 6-OHDA. Error bars = SEM of 3 biological replicates, each with 50–120 animals per strain and concentration. Total number of animals per condition n = 240–320 (n.s. p>0.05; G-Test). (C) Effect of ced-13 mutation on dopaminergic neurodegeneration after treatment with 0.75 mM 6-OHDA. Error bars = SEM of 3 biological replicates, each with 90–120 animals per strain and concentration. Total number of animals per condition n = 300–330 (n.s. p>0.05; G-Test). (TIF) [file pgen.1007106.s020.tif]

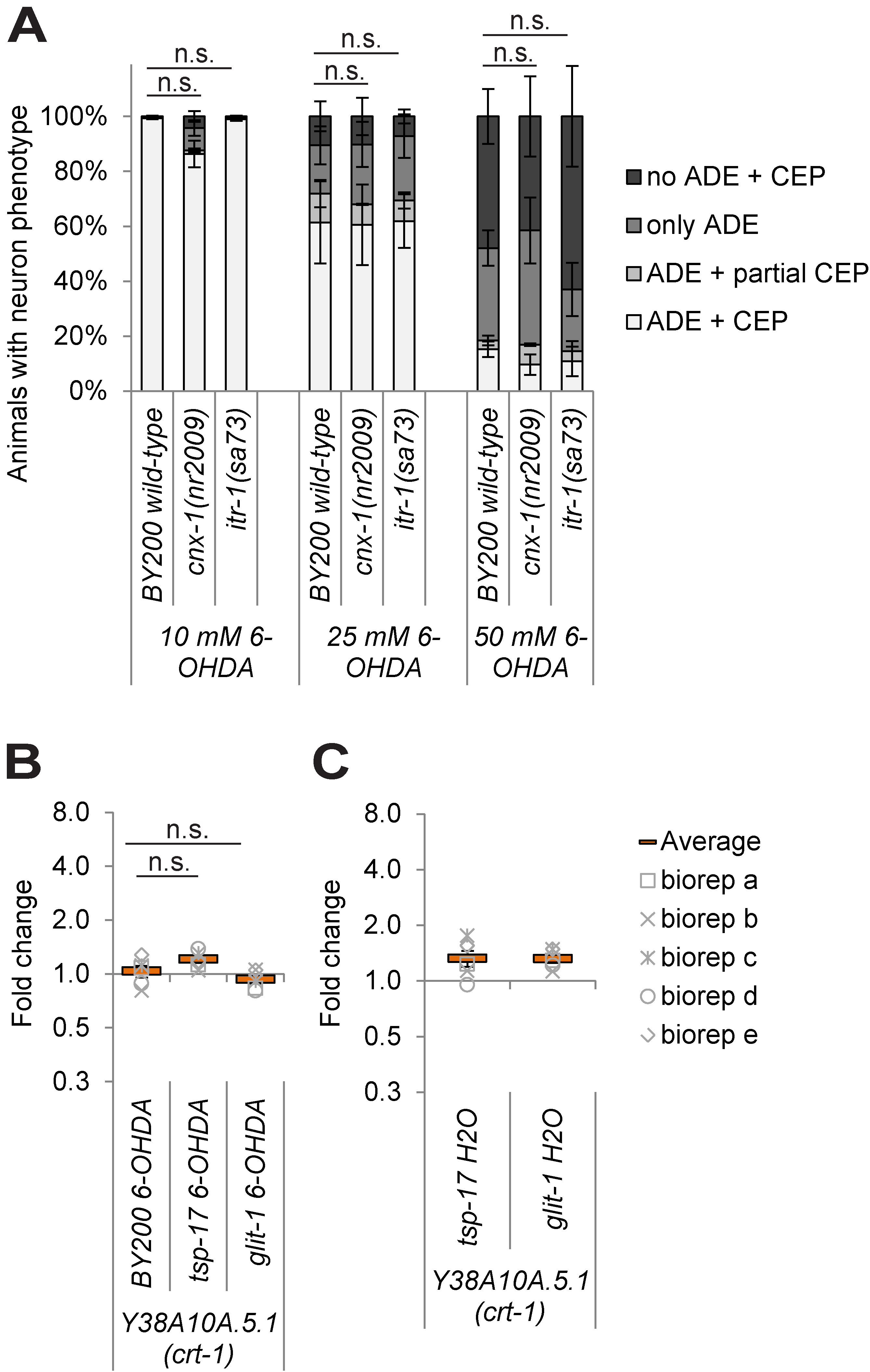

Supplement: S21 Fig — (A) Effect of cnx-1 and itr-1 mutations on dopaminergic neurodegeneration after treatment with 10, 25 and 50 mM 6-OHDA. Error bars = SEM of 3 biological replicates, each with 70–120 animals per strain and concentration. Total number of animals per condition n = 285–325 (n.s. p>0.05; G-Test). (B) crt-1 mRNA level analysis after 1h of 6-OHDA treatment in wild-type and mutant L1 stage larvae. (C) crt-1 mRNA level analysis in mutant L1 stage larvae as compared to wild-type animals under control conditions (treatment with H2O instead of 6-OHDA). (B) and (C) The data are normalised to the control gene pmp-3. The average and the respective values for 5 biological replicates (biorep a-e) are indicated. Error bars = SEM of 5 biological replicates (n.s. p>0.05; two-tailed t-test). (TIF) [file pgen.1007106.s021.tif]
