# Supplementary material for: Mutations in Caenorhabditis elegans neuroligin-like glit-1, the apoptosis pathway and the calcium chaperone crt-1 increase dopaminergic neurodegeneration after 6-OHDA treatment
Source: PLoS Genet. 2018 Jan 18;14(1):e1007106. doi: 10.1371/journal.pgen.1007106 (PMC5773152; doi:10.1371/journal.pgen.1007106)
Supplement: S1 Text — (PDF) [file pgen.1007106.s023.pdf]

S1 Text. Genetic interactions between *glit-1* and dopamine metabolism

(A) In *C. elegans*, dopamine can bind to D_1-type (stimulatory) dopamine receptor DOP-1 and the D_2-type (inhibitory) dopamine receptors DOP-2 and DOP-3 after release into the synaptic cleft (simplified cartoon in S11F Fig). We found that mutation of *dop-1* and *dop-3* did not alter 6-OHDA sensitivity, while mutation of *dop-2* led to a slight reduction of 6-OHDA-induced dopaminergic neurodegeneration in *glit-1* mutants (S11A Fig). Furthermore, *dop-2* mutation also decreased dopaminergic neuron loss in wild-type animals (S11 D, E Fig) [13] and *tsp-17* mutants [13]. In summary, the rescue of dopamine neuron loss by *dop-2* mutation is only partial and occurs in all tested genetic backgrounds, indicating that the function of *glit-1* is largely unrelated to *dop-2*. (B) Dopamine is synthesised de novo by CAT-2 (abnormal catecholamine distribution), a dopamine synthesis-specific tyrosine hydroxylase, and the BAS-1 aromatic amino acid decarboxylase, and packed into vesicles by the CAT-1 vesicular monoamine transporter (S11F Fig) [23]. We found that while *glit-1* mutant sensitivity was not modulated by *bas-1* mutation, dopaminergic neurodegeneration of *glit-1* mutants was increased by *cat-1* and *cat-2* mutations (S11B, C Fig). In contrast, in a wild-type background *cat-1* mutation decreased dopaminergic neuron loss and *cat-2* mutation did not show an effect (S11D, E Fig). Mutation of CAT-2, the key dopamine synthesis enzyme, is expected to cause reduced dopamine production. As dopamine and 6-OHDA likely compete for uptake via the dopamine transporter, it is conceivable that overall decreased dopamine levels cause increased neuronal uptake of 6-OHDA, and vice versa. Thus, a *cat-2* mutation might lead to increased 6-OHDA uptake into dopaminergic neuron, leading to increased cellular damage. In line with this argument, we and others reported previously that in the opposite scenario, overexpression of CAT-2 decreased 6-OHDA-induced dopaminergic neurodegeneration [13,62]. We speculate that *cat-2* deletion might cause different effects in *glit-1* mutants and wild-type animals due to the different concentrations of 6-OHDA used to analyse phenotypes in the respective background: at 0.75 and 10 mM 6-OHDA the detrimental effects of decreased intracellular dopamine levels might be still detectable, whereas at 25 and 50 mM 6-OHDA these effects might not make a difference

anymore.. We cannot explain why mutation of BAS-1 does not lead to similar effects as mutation of CAT-2; however, we note that only CAT-2, but not BAS-1, is specifically involved in dopamine synthesis [23]. Mutation of the vesicle-packing enzyme CAT-1 is in contrast expected to lead to increased cytosolic dopamine. Increased intracellular dopamine levels were suggested to be detrimental for dopaminergic neurons (for review see [63]). We speculate that after exposure of *cat-1* single mutants to high concentrations of 6-OHDA (25 mM and 50 mM), high levels of cytosolic dopamine might buffer the (even more) damaging effects of the drug.

62. Cao S, Gelwix CC, Caldwell K a, Caldwell G a. Torsin-mediated protection from cellular stress in the dopaminergic neurons of Caenorhabditis elegans. J Neurosci. 2005;25: 3801–12. doi:10.1523/JNEUROSCI.5157-04.2005

63. Caudle WM, Colebrooke RE, Emson PC, Miller GW. Altered vesicular dopamine storage in Parkinson’s disease: a premature demise. Trends Neurosci. 2008;31: 303–308. doi:10.1016/j.tins.2008.02.010
